# Anomalous Temperature Interdicts the Reproductive Activity in Fish: Neuroendocrine Mechanisms of Reproductive Function in Response to Water Temperature

**DOI:** 10.3389/fphys.2022.902257

**Published:** 2022-05-24

**Authors:** Md. Mahiuddin Zahangir, Mohammad Lutfar Rahman, Hironori Ando

**Affiliations:** ^1^ Marine Biological Station, Sado Island Center for Ecological Sustainability, Niigata University, Sado, Japan; ^2^ Department of Fish Biology and Biotechnology, Faculty of Fisheries, Chattogram Veterinary and Animal Sciences University, Chattogram, Bangladesh; ^3^ Department of Genetics and Fish Breeding, Faculty of Fisheries, Bangabandhu Sheikh Mujibur Rahman Agricultural University, Gazipur, Bangladesh

**Keywords:** GnIH, GnRH, grass puffer, hypothalamus, kisspeptin, reproduction, transient receptor potential, pituitary

## Abstract

Fish are poikilotherm and small changes in water temperature can greatly affect physiological processes including reproduction, which is regulated by complex neuroendocrine mechanisms that respond to climatic events. This review provides evidence that anomalous high and low temperature may directly affect reproduction in fish by suppressing the expression of genes in the reproductive neuroendocrine system. The grass puffer, *Takifugu alboplumbeus*, is an excellent animal model for studying the thermal regulation of reproduction, for they exhibit periodic spawning activities, which are synchronized with seasonal, lunar and daily cycles. In the grass puffer, the expression of the genes encoding gonadotropin-releasing hormone (GnRH) 1, kisspeptin, gonadotropin-inhibitory hormone (GnIH) and their receptors were markedly suppressed in the diencephalon of fish exposed to high temperature (28°C) when compared to normal temperature (21°C), followed by the decrease in the pituitary mRNA levels for follicle-stimulating hormone (FSH), luteinizing hormone (LH) and growth hormone (GH). On the other hand, the exposure to low temperature (14°C) also inhibited the expression of *gnrh1, kiss2, gnih* and their receptor genes in the brain and *fshb*, *lhb*, *gh* and *prl* in the pituitary. Taken together, it is plausible that anomalous high and low temperature may be a proximate driver of termination of reproduction by suppressing the activity of the reproductive GnRH/kisspeptin/GnIH system, possibly through direct action of temperature signals at transcription level.

## Introduction

The world’s climate has changed drastically over the last few centuries and considered as a crucial challenge to the humanity in the 21st century. At present, climate change is contributing to extreme climatic events that include heat waves, heavy rainfall and drought in a more frequent and severe fashion. While climate change is a global phenomenon, its negative effect falls upon in all living being, especially in the wild environment. Changes in the environmental factors (temperature, photoperiod, humidity, etc.) greatly influence on the physiology, behavior and ecology of a wide spectrum of organisms (Wuethrich, 2001; Walther et al., 2002). Indeed, an important biological function that has been affected by the climate change is reproduction; changes in the ambient temperature most likely leads to the advancement or delay in the reproductive events of seasonal breeders, particularly in temperate zone and this may cause a decrease in the wild resources because there is only a short period in the annual cycle when conditions are most suitable for reproduction of many seasonal breeders including fish (Wang et al., 2010).

Intragovernmental Panel on Climate Change (IPCC) is now devastatingly convincing that climate change is unpredicted, widespread, rapid and intensifying in upcoming days. IPCC also predicted that global surface temperature will increase 1.5°C above pre-industrial levels by 2030–2050 (Global Warming of 1.5°C, IPCC, 2021) and this may have a great impact on the biodiversity, ecosystems and livelihood, unless massive reduction in the CO_2_ and other greenhouse gases. So, it is high time to focus on the effect of climate change and the possible impact on the biodiversity of the planet.

The impact of climate change on the aquatic life broadly classified as direct and indirect. Rising water temperature, salinity, water acidification and transformed hydrological regimes are all direct effects. The indirect effects include the potential loss of estuarine habitat due to rising sea level as well as construction of dams and water abstraction for other purposes which limits the diversity and distribution of the wild fisheries resources. The effect of such environmental changes has a great impact of reproduction in fish (Servili et al., 2020). Among these factors, water temperature is a fundamental physical regulatory factor in the lives of fish and its effect is expressed strongly in the control of all reproductive and developmental processes including gamete development and maturation, ovulation and spermiation, spawning, embryogenesis, hatching, larval development and juvenile survival (Alix et al., 2020). Warmer waters decreased the biomass production (van Dorst et al., 2019), reducing the population structure which ultimately leads to the extinction of the species. For the protection, enhancement and restoration of the wild fisheries resources, it is necessary to understand the adaptive mechanism of wild species for water temperature and appropriate management strategies should be implemented for reducing the impact of global warming on individual species, ecosystems and society. As fish are poikilotherm, small changes in water temperature can greatly affect their physiological process which makes them an ideal animal model for studying the thermal regulation of reproduction.

## The Hypothalamo-Pituitary-Gonadal Axis in the Regulation of Reproduction

Reproduction in fish is controlled by the action of multiple endocrine and neuroendocrine factors in the hypothalamo-pituitary-gonadal (HPG) axis. The timing and success of reproduction is under the control of complex interaction between various environmental factors (temperature, photoperiod, moon cycle, tides, etc.) and multiple hypothalamic neurohormones including gonadotropin-releasing hormone (GnRH), kisspeptin, and gonadotropin-inhibitory hormone (GnIH) (Simonneaux et al., 2013; Dufour et al., 2020). Among these factors, GnRH is the principal regulator of the secretion of gonadotropins (GTHs), namely follicle-stimulating hormone (FSH) and luteinizing hormone (LH), from the pituitary in many vertebrate species including fish (Ando and Urano, 2005; Zohar et al., 2010; Muñoz-Cueto et al., 2020). Kisspeptin plays an important role in the neuroendocrine regulation of reproduction through stimulating the GnRH secretion in mammals (Oakley et al., 2009). In fish, there are two paralogous genes for kisspeptin (*kiss1* and *kiss2*) and kisspeptin receptor (*kissr1*, *kissr2*, *kissr3* and *kissr4*) and this increases the complexity of the kisspeptin system in fish (Ogawa and Parhar, 2018; Dufour et al., 2020). Although kisspeptin has also been shown to play an important role in fish reproduction, some authors described as stimulatory, inhibitory or having no effect on reproduction depending on the species (Felip et al., 2009; Kitahashi et al., 2009; Pasquier et al., 2011; Kanda et al., 2013; Nakajo et al., 2017; Ohga et al., 2018). The role of GnIH is to inhibit the GTH secretion by antagonistic interaction with GnRH in mammals and birds (Tsutsui et al., 2012; Ubuka et al., 2016). However, in fish, both stimulatory and inhibitory effects of GnIH on the secretion of GTH has been found depending on species and gonadal stage (Muñoz-Cueto et al., 2017; Di Yorio et al., 2019).

These hormonal inputs from the hypothalamus stimulate the synthesis and release of FSH and LH, which are pituitary glycoprotein hormones stimulating the gonadal maturation and the production of sex steroid hormones (Yaron et al., 2003). In addition, growth hormone (GH) and prolactin (PRL) have been shown to be involved in teleost’s gonadal development, gametogenesis, and steroidogenesis (Singh et al., 1988; Canosa et al., 2007; Whittington and Wilson, 2013). Previous studies in temperate fish species have shown that neuroendocrine components of the HPG axis exhibit substantial seasonality in their expression levels in association with changes in the environmental factors, especially water temperature. Here in this review, we focused on the effect of anomalous temperature on fish reproduction and the possible neuroendocrine mechanism in response to variable water temperature.

## Effects of Water Temperature on the Reproductive Neuroendocrine System in Fish

Water temperature has a remodelling role for fine tuning the reproductive cycle, that is, the precise timing of gamete formation, maturation and spawning, in particular environment (Van Der Kraak and Pankhurst, 1997; Pankhurst and King, 2010). To evaluate the effect of fluctuating water temperature on the reproductive events, a large number of experiments have been conducted in various teleost fishes. The effects of anomalous high and low temperature on the expression of the genes for various endocrine and neuroendocrine factors in the HPG axis have been examined in some teleost fish species and those on the hypothalamic neuropeptides and their receptors are summarized in Table 1 and those for the pituitary hormones and neurohormone receptors are summarized in Table 2. In the brain, it is evident that fluctuating water temperature reduced the expression levels of the genes for GnRH, kisspeptin, GnIH and their receptors, with the exception of GnRH3 and GnIH in sheepshead minnow and Kiss1 in zebrafish (Table 1). There are three GnRH isoforms in teleosts, namely GnRH1, GnRH2 and GnRH3, and their brain distribution and functional roles are different (Zohar et al., 2010; Muñoz-Cueto et al., 2020). Among them, preoptic GnRH1 or GnRH3 neurons, depending on fish species that possess two or three GnRH isoforms in their brains, directly innervate the pituitary and stimulate GTH secretion, and the gene expression of these GnRH isoforms were suppressed by anomalous high and low temperature in most cases (Table 1). In sheepshead minnow, the increased *gnrh3* expression of the fish exposed to high temperature conditions with reduced pituitary *fshb* and *lhb* expressions suggests that GnRH3 does not have the hypophysiotropic role in this fish (Bock et al., 2021). On the other hand, GnIH, which is also stimulated in the high temperature conditions, is considered to have a negative role in GTH secretion in sheepshead minnow (Bock et al., 2021). However, GnIH has been shown to have a stimulatory role in GTH synthesis in some fish species including the grass puffer, *Takifugu alboplumbeus*, in which the *gnih* expression was suppressed in the fish exposed to high temperature conditions (Table 1). In zebrafish, Kiss1 neurons are exclusively localized in the habenula and implicated in the modulation of the serotonergic system (Ogawa et al., 2012; Ogawa and Parhar, 2018), while Kiss2 neurons in hypothalamic nuclei are implicated in reproduction (Servili et al., 2011). Therefore, *kiss1*/*kissr1* expressions stimulated by low temperature was considered to be involved in the alteration of behavior under low temperature conditions in zebrafish (Shahjahan et al., 2013).

**TABLE 1:** Effects of water temperature on the brain of fish.

Species	Temperature treatment^a^	Exposure Time	Gene expression		References
			Stimulation	Suppression	
Effect of high Temperature					
Red seabream (*Pagrus major*)	24°C (+7°C)	19 days		GnRH1	Okuzawa et al. (2003)
Red seabream (*Pagrus major*)	24°C (+7°C)	5 and 10 days		GnRH1	Okuzawa and Gen, (2013)
Blue gourami (*Trichogaster trichopterus*)	31°C (+4°C)	9 days		GnRH3, IGF-1	David and Degani, (2011)
Blue gourami (*Trichogaster trichopterus*)	31°C (+4°C)	9 days		GnRH3, PACAP	Levy et al. (2011)
Pejerrey (*Odontesthes bonariensis*)	27°C (+8°C)	8 days		GnRH1	Elisio et al. (2012)
Zebrafish (*Danio rerio*)	35°C (+8°C)	7 days		GnRH3, Kiss2, Kissr2^b^	Shahjahan et al. (2013)
Grass puffer (*Takifugu alboplumbeus*)	28°C (+7°C)	7 days		GnRH1, Kiss2, Kiss2R	Shahjahan et al. (2017)
Grass puffer (*Takifugu alboplumbeus*)	28°C (+7°C)	7 days		GnIH, GnIH-R	Rahman et al. (2019)
Sheepshead minnow (*Cyprinodon variegatus*)	37°C (+10°C)	14 days	GnRH3, GnIH	Isotocin	Bock et al. (2021)
Effect of low temperature					
Blue gourami (*Trichogaster trichopterus*)	23°C (–4°C)	9 days		GnRH3, PACAP, IGF-1	David and Degani, (2011)
Blue gourami (*Trichogaster trichopterus*)	23°C (–4°C)	9 days		GnRH3, PACAP, IGF-1	Levy et al. (2011)
Zebrafish (*Danio rerio*)	15°C (–12°C)	7 days	Kiss1, Kissr1, Kissr2^c^	GnRH3, Kiss2, Kissr2^b^	Shahjahan et al. (2013)
Grass puffer (*Takifugu alboplumbeus*)	14°C (–7°C)	7 days		GnRH1, Kiss2, Kiss2R	Shahjahan et al. (2017)
Grass puffer (*Takifugu alboplumbeus*)	14°C (–7°C)	7 days		GnIH, GnIH-R	Rahman et al. (2019)

a The temperature on the left side of parentheses indicates anomous temperature in which the fish were treated. The temperature in parentheses indicates differene between the anomous temperature and normal temperature.

b *kissr2* mRNA levels in the the caudal zone of the periventricular hypothalamus and the posterior tuberal nucleus.

c *kissr2* mRNA levels in the nucleus of the medial longitudinal fascicle, oculomotor nucleus and the interpeduncular nucleus.

**TABLE 2:** Effects of water temperature on the pituitary of fish.

Species	Temperature treatment^a^	Exposure time	Gene expression		References
			Stimulation	Suppression	
Effect of high temperature					
Red seabream (*Pagrus major*)	24°C (+7°C)	19 days		GnRHR, LHβ	Okuzawa et al., 2003
Red seabream (*Pagrus major*)	24°C (+7°C)	5 and 10 days		GnRHR, FSHβ, LHβ	Okuzawa and Gen, 2013
Pejerrey (*Odontesthes bonariensis*)	27°C (+8°C)	8 days		LHβ	Soria et al., 2008
Pejerrey (*Odontesthes bonariensis*)	27°C (+8°C)	8 days		GPHα, FSHβ, LHβ	Elisio et al., 2012
Blue gourami (*Trichogaster trichopterus*)	31°C (+4°C)	9 days		LHβ, PRL	David and Degani, 2011
Blue gourami (*Trichogaster trichopterus*)	31°C (+4°C)	9 days		FSHβ, LHβ, GH	Levy et al., 2011
European eel (*Anguilla*)	20°C (+10∼+2°C)	12 weeks		FSHβ, LHβ	Pérez et al., 2011
Grass puffer (*Takifugu alboplumbeus*)	28°C (+7°C)	7 days		FSHβ, LHβ	Shahjahan et al., 2017
Grass puffer (*Takifugu alboplumbeus*)	28°C (+7°C)	7 days		GH	Rahman et al., 2019
Sheepshead minnow (*Cyprinodon variegatus*)	37°C (+10°C)	14 days		FSHβ, LHβ, TSHβ	Bock et al., 2021
Effect of low temperature					
Blue gourami (*Trichogaster trichopterus*)	23°C (–4°C)	9 days		FSHβ, LHβ, PRL	David and Degani, 2011
Blue gourami (*Trichogaster trichopterus*)	23°C (–4°C)	9 days		FSHβ, LHβ, GH	Levy et al., 2011
Grass puffer (*Takifugu alboplumbeus*)	14°C (–7°C)	7 days		FSHβ, LHβ	Shahjahan et al., 2017
Grass puffer (*Takifugu alboplumbeus*)	14°C (–7°C)	7 days		GnIH-R, GH, PRL	Rahman et al., 2019

a The temperature on the left side of parentheses indicates anomous temperature in which the fish were treated. The temperature in parentheses indicates differene between the anomous temperature and normal temperature.

In the pituitary, the expression of the genes for GTH subunits, GH, PRL as well as receptors for GnRH and GnIH was significantly suppressed by anomalous high and low temperature in all cases examined (Table 2). These changes have been shown to be accompanied with the inhibition of oogenesis and steroidogenesis and termination of reproduction. Taken together, these results suggest that anomalous temperature mostly has an inhibitory effect on fish reproduction by suppressing the activity of the reproductive GnRH/kisspeptin/GnIH system, possibly through direct action of temperature signals at transcription level.

## Reproductive Periodicity and Changes in the Expression of the Reproductive Neuroendocrine System in the Grass Puffer

To address the molecular neuroendocrine regulation of reproductive periodicity and temperature regulation of reproduction, the grass puffer provides an excellent animal model because grass puffer displays a strong reproductive periodicity. The grass puffer is a common intertidal puffer species in the Northwest Pacific Ocean, ranging from as far north as Japan down to the Philippines (Shao et al., 2014). In Japan, grass puffer usually inhibits environments between 10 and 29°C and spawning occurs during spring to early summer with ambient water temperature ranging from about 17 to 25°C. Interestingly, the timing of spawning is tightly connected with the lunar and diurnal cycles during the spawning season (Motohashi et al., 2010; Ando et al., 2013, 2018). During spring tide, on the new and full moon days, the fish aggregate at certain seashore places for spawning and spawning starts 1.5–2.0 h before high tide and continues for about 1 h during the rising tidal phase. Therefore, the spawning of grass puffer is tightly connected to the seasonal, lunar, daily and tidal rhythms where the environmental factors, such as water temperature, light (daylight and moonlight) and tidal cycle may interplay in the control of reproduction.

We previously examined the expression of the genes for the hypothalamic neurohormones and their receptors in the brain of grass puffer over several months during the reproductive cycle. We developed a quantitative real-time PCR assay for each mRNA (Motohashi et al., 2008; Shahjahan et al., 2010a, 2010b, 2011), which is so reliable to generate the sensitivity of 100 copies of particular mRNA with satisfactory range of variation. In the brain of grass puffer, three forms of GnRHs displayed differential expression patterns throughout the spawning season. The expression levels of *gnrh1* and *gnrh3* were significantly elevated during spawning season, while *gnrh2* does not show any variations (Shahjahan et al., 2010a). The genes encoding kisspeptin (*kiss2*) and its receptor (*kiss2r*) was predominantly expressed over the course of sexual maturation from the pre-spawning to post-spawning stages (Shahjahan et al., 2010b). On the other hand, mRNA levels for GnIH (*gnih*) and its receptor (*gnihr*) gradually increased during the spawning period and significantly decreased at the post-spawning stage (Shahjahan et al., 2011).

In the pituitary, the expression levels of GTH subunit genes (*gpa, fshb* and *lhb*) were extensively increased in parallel with *gnrh1* and *gnrh3* during the spawning season (Shahjahan et al., 2010a). The expression of *gh* was also significantly higher in the spawning and post-spawning stages, while the expression of *prl* was significantly higher in the pre-spawning and spawning stages (Shahjahan et al., 2016). Moreover, the plasma concentration of estradiol-17β (E2) and testosterone (T) were higher in the mature fish at the spawning stage compared to other reproductive stages (Shahjahan et al., 2010a). In addition, these genes demonstrated diurnal and circadian oscillations in their expression levels during the spawning period (Shahjahan et al., 2011; Ando et al., 2014).

Tiger puffer, *Takifugu rubripes*, is closely related to grass puffer, also displayed reproductive periodicity in the expression of the HPG axis genes. The mRNA levels of three *gnrhs*, *gnih*, *gnihr*, *fshb* and *lhb* were extensively higher in the mature fish compared to the immature fish, especially in females, and the expression of these genes was significantly decreased in the post-ovulatory females (Zahangir et al., 2021). So, grass puffer as well as the closely related tiger puffer displayed a strong reproductive periodicity with the dynamic variation in the expression of the hypothalamic neurohormone and pituitary hormone genes.

## Effects of Anomalous High Temperature on the Expression of the Reproductive Neuroendocrine System in the Grass Puffer

**FIGURE 1:**
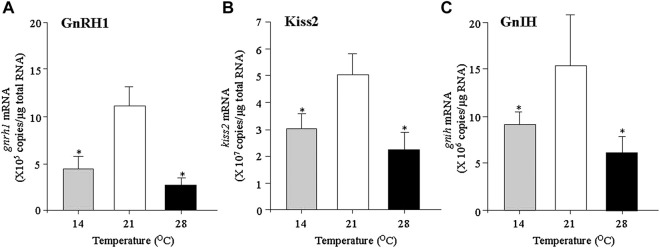
Changes in the expression levels of *gnrh1* **(A)**, *kiss2* **(B)** and *gnih* **(C)** in the diencephalon of grass puffer exposed to low temperature (14°C), normal temperature (21°C) and high temperature (28°C) conditions for 7 days. Values are presented as mean ± standard error of the mean (SEM) (n = 8 in each group). Asterisk indicates a significant difference in the low and high temperature groups compared to the normal temperature group (*, *p* < 0.05).

In our previous studies, the mature grass puffer was exposed to a normal temperature (21°C), low temperature (14°C) and a high temperature (28°C) for 7 days, and the expression levels of the HPG axis genes were measured. The mRNA levels of *gnrh1*, *kiss2* and *gnih* in the diencephalon were significantly decreased in the fish exposed to high temperature compared to normal temperature (Figure 1). In addition, the expression levels of *kiss2r* and *gnihr* were significantly decreased in the high temperature-exposed fish (Shahjahan et al., 2017; Rahman et al., 2019). In the pituitary, the exposure to high temperature significantly decreased the levels of *gnihr* mRNA as well as *fshb*, *lhb* and *gh* mRNAs (Shahjahan et al., 2017). In the grass puffer, GnIH has been shown to be a multifunctional hypophysiotropic factor that stimulates the expression of not only *fshb* and *lhb* but also *gh* and *prl* (Shahjahan et al., 2011, 2016; Ando et al., 2018). Taken together, the findings on the grass puffer indicate that anomalous high temperature (28°C) has an inhibitory effect on the GnRH1/Kiss2/GnIH/GTH/GH system and this may lead to termination of reproduction at the end of breeding season in July in nature.

## Effects of Anomalous Low Temperature on the Expression of the Reproductive Neuroendocrine System in the Grass Puffer

Our previous studies revealed that the mRNA levels of *gnrh1*, *kiss2*, *kiss2r*, *gnih* and *gnihr* in the diencephalon were significantly decreased in the low temperature (14°C) conditions when compared to the normal temperature (21°C) conditions (Shahjahan et al., 2017; Rahman et al., 2019). At the pituitary level, the mRNA levels of *fshb*, *lhb*, *gh* and *prl* were significantly decreased in the low temperature conditions.

Since fish are poikilotherm, they cannot circulate the heated blood throughout the body, hence low temperature may be highly susceptible as stress (Donaldson et al., 2008). The response to stress is delineated by the incitement from the brain and the release of stress hormone like cortisol and catecholamine results in the activation of the neuroendocrine system and a consequential cascade for the metabolic and physiological changes (Zahangir et al., 2015). In the grass puffer exposed to the anomalous low temperature (14°C) conditions, the plasma levels of cortisol were significantly elevated compared to the normal temperature (21°C) conditions, whereas no noticeable change was observed in the high temperature (28°C) conditions, suggesting that the high temperature conditions are within the physiologically acceptable level (Shahjahan et al., 2017). It is therefore considered that the inhibition of *prl* expression observed only in the low temperature conditions was most probably due to stress response. The inhibitory action of cortisol on the *prl* expression in the tilapia pituitary supports this notion (Uchida et al., 2004).

## Perspectives: Transient Receptor Potential Channels as Possible Molecular Signals of Temperature on the Reproductive Neuroendocrine System

The molecular mechanism of thermal signal transduction in the reproductive neuroendocrine system in response to variable water temperature is largely unknown. Teleost fish have been suggested to sense water temperature using surface thermal receptors located in the trunk lateral line (Sullivan, 1954), and those in trigeminal ganglia have been shown to be involved in thermoregulatory behavior (Haesemeyer, 2020). Although there is a paucity of information concerning on the neural circuits involved in the temperature-dependent regulation of the HPG-axis, temperature is primarily detected by thermo-gated ion channels in somatosensory neurons (Gracheva and Bagriantsev, 2015). It is well known in vertebrates that thermosensitive transient receptor potential (TRP) ion channels called thermoTRP channels play an important role in transducing temperature signals to neural signals (Patapoutian et al., 2003; Diaz-Franulic et al., 2016). Among thermoTRP family members such as ankyrin TRP (TRPA), metastatin TRP (TRPM) and vanilloid TRP (TRPV), TRPV1 and TRPA1 sense noxious ranges of hot and cold stimuli, respectively (Saito and Tominaga, 2017). Although thermoTRPs are mainly expressed in primary sensory neurons, TRPV has also been shown to express in the central nerve system in mammals, with widespread and discrete brain regions including the hypothalamus (Cavanaugh et al., 2011; Nedungadi et al., 2012). Regarding the HPG axis, canonical TRP (TRPC) is expressed in GnRH and kisspeptin neurons (Götz et al., 2017). TRPC channels in mouse GnRH neurons are involved in the kisspeptin-induced depolarization (Zhang et al., 2008) and the expression of TRPC channel subunits are upregulated by estrogen, suggesting that TRPC plays a role in reproductive stage-dependent stimulation of GnRH neurons by kisspeptin (Bosch et al., 2013).

**FIGURE 2:**
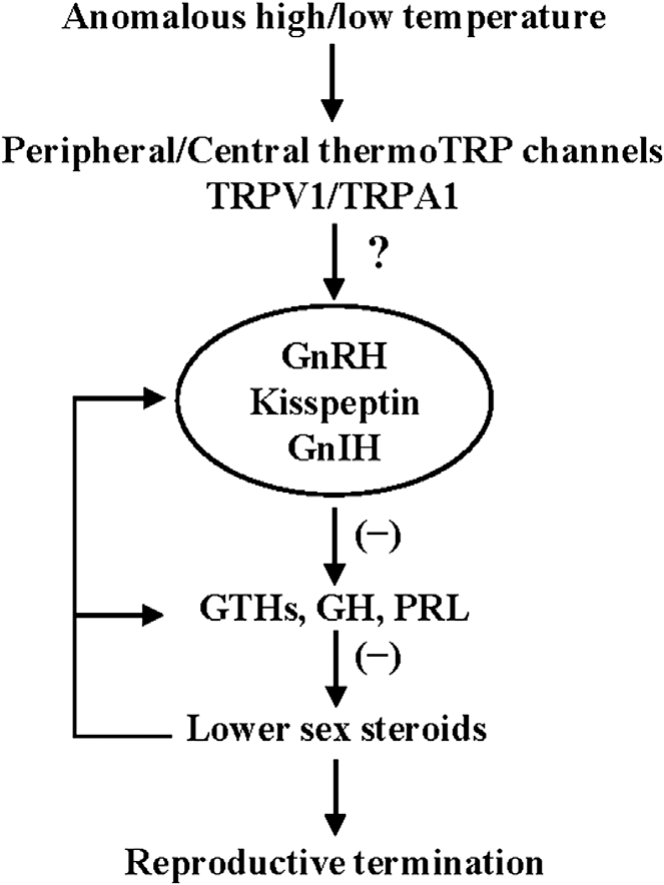
Schematic representation of the proposed neuroendocrine mechanism of thermosensitive regulation of reproduction in fish. See the text for explanation.

In fish, TRPV members (V1-V4) has been shown to be activated by noxious high temperature (Dhaka et al., 2006). In salmonids, TRPV1 and TRPV4 are expressed in the pineal photoreceptor cells and may mediate the effects of temperature on melatonin production (Nisembaum et al., 2022). On the other hand, the thermal sensitivity of TRPA1 is variable among vertebrate species (Saito and Tominaga, 2017). The zebrafish TRPA1 was shown to respond to both heat and cold stimuli when expressed in *Xenopus* oocytes (Oda et al., 2016). Since fish are poikilotherm, it is possible that thermoTRP channels in the brain may serve as a thermal sensor that directly senses variable water temperature and its signals may be transmitted to the hypothalamus. In fact, temperature-sensitive neurons have been identified in the hypothalamus of fish (Greer and Gardner, 1974; Nelson and Prosser, 1981). Further studies on the function and expression of thermoTRP channels in the peripheral somatosensory neurons and also in the hypothalamus would provide valuable information on the molecular mechanisms of thermosensitive regulation of reproduction in fish (Figure 2).

## Conclusion

The evidence summarized in this review revealed that anomalous temperature could directly affect the activity of the reproductive neuroendocrine system, inhibiting the expression of the genes for the hypothalamic neurohormones and their receptors and the pituitary hormones, i.e., the expression of *gnrh1/3*, *kiss2*, *kiss2r*, *gnih* and *gnihr* in the diencephalon and *fshb*, *lhb*, *gh* and *prl* in the pituitary. Grass puffer shows a strong reproductive periodicity where high water temperature (28°C) transmits environmental signals to inhibit the activity of the reproductive neuroendocrine system for the termination of breeding season. On the other hand, anomalous low temperature during the breeding season also transmits environmental signals to inhibit the activity of reproductive neuroendocrine system as, in part, a response to stress in the grass puffer. Taken as a whole, the reproductive neuroendocrine system is most probably the primary target of anomalous changes in water temperature and their effects are attributed to the transcriptional regulation of the genes for the hypothalamic neurohormones and their receptors and the pituitary hormones.
